# Experiences of functional recovery after polytrauma in a private Johannesburg healthcare setting: Patients’ perspectives

**DOI:** 10.4102/sajp.v82i1.2195

**Published:** 2026-05-28

**Authors:** Mughammad A. Reis, Monique M. Keller, Heleen van Aswegen

**Affiliations:** 1 Department of Physiotherapy, School of Therapeutic Sciences, Faculty of Health Sciences, University of the Witwatersrand, Johannesburg, South Africa; 2 Department of Physiotherapy, Faculty of Health Sciences, University of the Witwatersrand, Johannesburg, South Africa

**Keywords:** physiotherapy, polytrauma injury, patient’s perceptions, rehabilitation, South Africa, patient satisfaction, physical recovery

## Abstract

**Background:**

The prevalence of polytrauma is increasing globally and in South Africa, contributing to the reported rates of disability. Early physiotherapy intervention in the acute care setting reduces the risk of complications and facilitates functional recovery of patients who sustained polytrauma injuries. There is minimal evidence exploring patients’ perceptions of their recovery of physical function following polytrauma injury in an acute setting and after hospital discharge.

**Objectives:**

To explore patients’ perceptions of their recovery of physical function after polytrauma.

**Method:**

A qualitative single-case study was carried out at a private trauma facility in Johannesburg, drawing on eight semi-structured interviews gathered through purposive sampling. The interview transcripts were examined using an inductive analytical approach, with MAXQDA assisting in generating the codes and themes.

**Results:**

Six central themes emerged, capturing patients’ views on the factors that hindered or supported their recovery of physical function following polytrauma. These broad themes included satisfaction with the care received, experiences with rehabilitation after discharge, mental health and resilience, degree of disability, pain and physical functioning, and the influence of support systems.

**Conclusion:**

Multiple factors shape how patients perceive their physical recovery after experiencing polytrauma. Notably, the quality of interpersonal interactions played a prominent role in shaping their views of the care they received.

**Clinical implications:**

Our study may inform practice in the clinical setting in the management of patients recovering from polytrauma injuries to address barriers and promote facilitators identified.

## Introduction

Polytrauma is a life-threatening condition in patients who have sustained multiple injuries to various parts of the body because of physical trauma, resulting in orthopaedic and systemic injuries (Rau et al. 2017). Patients who survive traumatic events often do not receive the quality of care required to optimise their recovery (Van Breugel et al. 2020). Common causes of polytrauma locally include road traffic accidents (61%), falls from height (25%) and falling heavy objects (7%) (Arumugam et al. 2015). South Africa, an upper-middle-income country, experiences a high burden of traumatic injury, with approximately 50 000 trauma-related deaths annually (Hardcastle et al. 2016). Globally, the World Health Organization reports 5 million annual injury-related deaths, nearly one-fifth occurring in Africa (Mathers 2008). Trauma survivors in South Africa outnumber deaths by 10–50 times, with approximately 50% living with permanent disability (Matzopoulos et al. 2015).

In KwaZulu-Natal, for example, 10 644 trauma patients presented to hospital services in 1 year, with 35% as a result of motor vehicle accidents, 35% to assaults, 26% to stabbings and 5% to gunshot wounds (Moodley, Aldous & Clarke 2014). Trauma accounts for up to 25% of the public hospital workload, with 90% of cases occurring in low-income communities (Hardcastle et al. 2016). High-income countries generally have more structured trauma systems with established referral pathways and rehabilitation programmes, which reduce long-term disability (Van Breugel et al. 2020).

Polytrauma injuries, often sustained through high-energy mechanisms, frequently require hospitalisation and intensive care unit (ICU) admission for specialised management (Amato et al. 2021). Long-term immobility because of prolonged bed rest can lead to neuromuscular weakness, persistent physical impairments, loss of function and reduced quality of life (Aitken et al. 2016).

Studies have shown that up to 85% of polytrauma patients experience ongoing pain 5 years post-injury, with the severity of pain correlating to the severity of the initial trauma (Gross & Amsler 2011). Prolonged bed rest contributes to patient deconditioning, depression, lethargy and neuromuscular instability (Padovani et al. 2016). Early physiotherapy improves outcomes by reducing hospital length of stay and enhancing physical function (Frandsen et al. 2024). As survival rates increase, the resulting disabilities post-ICU admission have increased, including reduced mobility and limitations in activities of daily living (Iddagoda et al. 2024; Pape et al. 2010).

Recovery after polytrauma is influenced by several factors. Patients report taking ownership of their recovery when provided with guidance and support in hospital and post-discharge (Claydon, Robinson & Aldridge 2017).

Family support is also critical in shaping recovery perceptions (Boyle et al. 2018). Despite these supports, only a minority of patients return to work 1 year post-injury; Neubert et al. (2025) found a return-to-work rate of 32%, highlighting long-term functional and socio-economic consequences. Pain is a major barrier to recovery, influenced by physical, psychological and social factors (Ahmadi et al. 2025).

While research exists on quality of life post-polytrauma, few studies have specifically explored patients’ perceptions of care and recovery. For example, Bouman et al. (2017) conducted a study in the Netherlands and reported that patients valued personalised rehabilitation and clear communication with healthcare providers, but often experienced gaps in support post-discharge. Similar findings in high-income countries indicate that inadequate guidance and limited follow-up can negatively affect recovery and patient satisfaction (Neubert et al. 2025). However, there is limited evidence from low- and middle-income countries, including South Africa, where trauma systems are less structured, rehabilitation services are often constrained, and access to care varies substantially between public and private healthcare settings.

Private healthcare patients were selected for our study because they represent a distinct context within the South African health system, where patients generally have access to well-resourced trauma facilities and rehabilitation services, yet little is known about their lived experiences of recovery post-polytrauma. Investigating perceptions within this population allows the study to examine whether the availability of resources translates into improved patient experiences and recovery outcomes. Moreover, insights from private healthcare patients can identify gaps that may not be apparent in public healthcare settings, thereby informing strategies to optimise rehabilitation and support services across different healthcare contexts.

Prolonged hospitalisation and delayed rehabilitation increase financial burden on patients, families, society and the healthcare system (Carpenè et al. 2010; Seneff et al. 2000).

To address this gap, the present study explored patients’ perceptions of their recovery of physical function following polytrauma, focusing on in-hospital care, rehabilitation services, secondary complications, pain and support structures. Understanding patient experiences in a private healthcare setting can guide improvements in rehabilitation services, enhance outcomes and reduce the long-term socio-economic impact of trauma.

## Research methods and design

This qualitative study utilised a constructivist paradigm, underpinned by an ontological perspective that reality is socially constructed and understood through individuals’ experiences. In this context, our study explored patients’ perceptions of their recovery of physical function following polytrauma, focusing on how in-hospital care, rehabilitation services, pain management, secondary complications and support structures influenced their recovery. Semi-structured interviews were employed to allow participants to share detailed, personal accounts of their recovery journeys, providing rich insight into the factors they perceived as most important in regaining physical function.

Our study focused on patients’ perceptions of their health and rehabilitation care, which was crucial for the author in understanding the phenomenon of recovery of physical function following polytrauma. By exploring these perceptions, our study sought to uncover how patients experienced and interpreted the care they received, the challenges they faced, and the factors they considered most influential in their rehabilitation, thereby revealing the reality and presence of the phenomenon under investigation. Additionally, our study incorporated an epistemological perspective, enabling the author to articulate this population’s unique experiences of care.

Purposive sampling was employed to identify potential participants from the physiotherapy practice’s patient database. The inclusion criteria were clearly defined to ensure that only appropriate participants were recruited. Eligible participants were adults aged 18 years or older, of any gender, who had sustained multiple orthopaedic injuries to the upper and/or lower limbs, with or without additional trunk injuries, and who required admission to the ICU for treatment. This inclusion criterion was designed to focus on patients who experienced significant polytrauma, as these individuals are more likely to face complex recovery trajectories, including prolonged hospitalisation, intensive rehabilitation needs and potential secondary complications. Including only adults ensures that participants can provide informed consent and reliably report their perceptions and experiences of care and recovery. Intensive care unit admission was specifically included to capture patients with severe injuries requiring high-level care, as their recovery experiences are likely to be distinct from those with less severe trauma, making their perspectives particularly valuable for understanding the challenges of rehabilitation, physical function recovery, and the support structures needed in a private healthcare setting. Participants were required to have been discharged from the hospital for 6 months to 1 year to ensure sufficient time for typical healing processes to occur.

Individuals with isolated head injuries, spinal cord injuries, amputations or pre-existing cognitive impairments were excluded to ensure a more homogeneous sample and to maintain the integrity of the study’s focus on physical recovery following polytrauma. These conditions each involve distinct recovery trajectories, rehabilitation needs, and functional limitations that differ substantially from those associated with multiple orthopaedic injuries. Including such patients could have introduced significant variability, making it difficult to attribute perceptions of physical recovery specifically to polytrauma-related orthopaedic injuries. Additionally, cognitive impairments may affect a participant’s ability to provide reliable accounts of their experiences, thereby influencing the validity and consistency of the qualitative data. Physiotherapy clinicians initially screened their database to identify patients who met the inclusion criteria, drawing on their knowledge of each patient’s clinical background. Only after patients provided verbal permission for their contact information to be released were they approached by the principal investigator for recruitment and formal consent. This ensured that the selection process was based on clinical eligibility rather than patient availability or convenience, in line with the purposive sampling strategy.

The semi-structured interview schedule was developed based on a thorough review of existing literature on patient recovery and the factors influencing perceptions of care in polytrauma cases. Questions were designed to elicit in-depth responses regarding patients’ experiences, focusing on aspects such as satisfaction with care, rehabilitation processes and mental health.

During the interview procedure, participants were engaged in a conversational manner, allowing for follow-up questions and clarifications to deepen the exploration of their perceptions, which facilitated a richer understanding of their recovery journey. The interviews were audio-recorded and transcribed verbatim by a transcription service, after which the data were reviewed and cleaned up by the author.

An inductive thematic analysis was conducted using MAXQDA (version 2018.2) (VERBI GmbH, Berlin, Germany). The process began with the author thoroughly reading and becoming familiar with each interview transcript. From this immersion, initial broad codes were generated and subsequently organised into overarching themes, following the steps outlined by Braun and Clarke (2019). Codes and themes were developed after each interview to help determine when no new insights were arising, signalling data saturation. To enhance the trustworthiness of the analysis, an external reviewer independently co-coded the transcripts.

Several strategies were applied to ensure rigour in this qualitative study. Credibility was supported by maintaining consistent interview procedures and using the same approach to questions across all interviews. Transferability and dependability were strengthened through comprehensive documentation of the study’s methods. The author also kept a reflective journal to capture observations of non-verbal cues.

### Ethical considerations

Ethical considerations, or axiology, played a vital role in ensuring that our study refrained from making judgments about patients’ perceptions. The principal investigator subsequently contacted the patients to arrange interview times, after which electronic written informed consent was secured for both participation and audio recording. Ethical clearance to conduct our study was obtained from the Human Research Ethics Committee (Medical) of the University of the Witwatersrand (clearance number: R14/49; Protocol no. M200951). Participants were informed that their information would remain confidential, with all data stored on a password-protected computer accessible only to the authors. These procedures were carried out between September 2020 and February 2021.

## Results

Thirteen individuals were invited to take part in our study, and eight agreed to participate. All eight semi-structured interviews; conducted in English, was the participants’ home language in our study; and were held via video platforms such as WhatsApp, Skype or Zoom, depending on each participant’s preference. Each interview lasted approximately 30 min. While virtual interviews inherently limit the authors’ ability to observe full body language, facial cues and expressions were still noted.

Participants ranged in age from 21 years to 59 years. One quarter of the sample consisted of women, while the remaining 75% were men. Most participants had been discharged for between 6 months and 9 months at the time of their interview. Hospital stays varied widely, from under 1 month to as long as 6 months, with male participants generally experiencing longer admissions. All participants had sustained their injuries as a result of motor vehicle accidents (see Table 1).

**TABLE 1:** Patient demographics.

Participant number	Age (years)	Gender	Mechanism of injury	Length of stay range (months)
1	56	Female	Motor vehicle accident	1–3
2	59	Male	Motor vehicle accident	< 1
3	43	Male	Motor vehicle accident	3–6
4	21	Male	Motor vehicle accident	< 1
5	28	Male	Motor vehicle accident	1–3
6	39	Female	Motor vehicle accident	< 1
7	19	Male	Motor vehicle accident	1–3
8	45	Male	Motor vehicle accident	1–3

**FIGURE 1:**
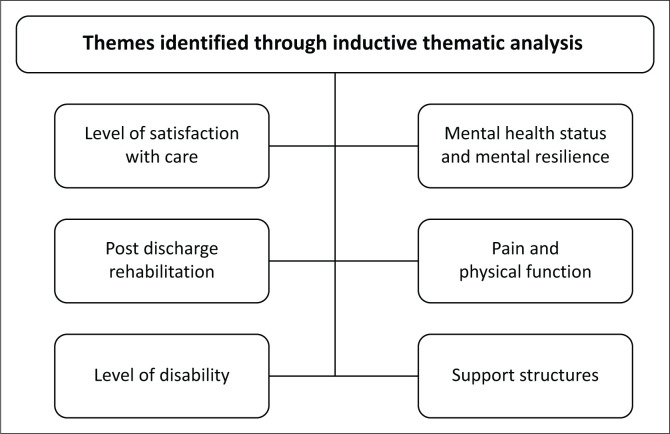
Overview of the current study’s findings, highlighting the key themes.

Through inductive thematic analysis, six themes were identified and later organised under each objective of the current study during the write-up. The overarching themes include: level of satisfaction with care, post discharge rehabilitation, mental health status and mental resilience, level of disability, pain and physical function and support structures. These themes are illustrated in Figure 1, followed by a detailed discussion of each theme.

### Level of satisfaction with care

The overarching theme from participants’ experiences of in-hospital care was that their satisfaction with the care received influenced their recovery after injury. Subthemes included the quality of care, communication and emotional support. Most participants felt that care was of a high standard, the hospital environment was clean and staff were professional, although some reported that night-shift staff were loud and unprofessional, disturbing their sleep. Poor communication with hospital staff was frequently noted, with some participants feeling that physiotherapy was overly demanding and not tailored to their needs. Participants also reported a lack of emotional support and a strong desire to return to a familiar environment. This is presented in Table 2:

**TABLE 2:** Participant responses relating to satisfaction with level of care received during their hospital stay. Overarching theme: Level of satisfaction with care influencing physical function recovery.

Sub-themes	Quotes
Progression of rehabilitation	‘The physios – changing of personnel was a challenge …’ (Participant 2, 59 years old, male, less than 1 month’s stay)
Emotional needs	‘There was no psychologist referral during hospital stay …’ (Participant 3, 43 years old, male, 3–6 months’ stay)
Quality of care	‘They did not change their methodology or the approach to my rehab program. It was basically a one size fits all approach which didn’t suit me and the type of injury I had.’ (Participant 3, 43 years old, male, 3–6 months’ stay)
Medical staff support	‘The nurses were very caring.’ (Participant 3, 43 years old, male, 3–6 months’ stay)
Poor communication	‘They told me we’re going to go on this date and somewhere there was a communication gap and I only got to go a week later.’ (Participant 5, 28 years old, male, 1–3 months’ stay)
Negative perceptions of physiotherapy	‘There was one physio that made me cry so badly that I asked her please stop. And she said no, she needs to go on. And I cried like a little baby.’ (Participant 7, 19 years old, male, 1–3 months’ stay)
Positive experience	‘Everything that I did in physio helped yeah because they made me more stronger than I was before.’ (Participant 1, 56 years old, female, 1–3 months’ stay) ‘We … there I did this in the physiotherapists what was with me through the whole process. And so there I started to practice more with the leg … learning all of your daily moves … they really helped me with able to use the leg more.’ (Participant 2, 59 years old, male, less than 1 month’s stay)

### Post discharge care influencing physical function recovery

The overarching theme that arose from participants’ experiences of post discharge care is the influence rehabilitation service provision, or lack thereof, had on physical function recovery. The corresponding sub-themes which arose include the following: care at rehabilitation centres was noted to have greatly assisted with recovery of physical function (Participant 2).

In this context, ‘care’ refers to the structured therapeutic interventions provided by the rehabilitation healthcare providers, including physiotherapy and occupational therapy. The effectiveness of this care was influenced both by the type of care delivered, such as hands-on physiotherapy, mobility training and patient education, and by the frequency of attendance, with regular sessions contributing to more noticeable improvements in physical function.

Participants highlighted that regular engagement with therapy supported the gradual recovery of strength, mobility and independence in daily activities. A key limitation to post-discharge care, both at home and in rehabilitation centres, was financial constraints (Participants 1 and 3). High costs and job loss were also reported as barriers (Participants 1 and 7). As a result, participants often received only partial rehabilitation, leaving them motivated to continue therapy after noticing improvements in physical function and understanding their potential for further recovery (Participant 3). This information is summarised in Table 3.

**TABLE 3:** Participant responses relating to rehabilitation services after discharge encompassing financial constraints and the rehabilitation facility. Overarching theme: Post discharge rehabilitation influencing physical function recovery.

Sub-themes	Quotes
Home-based rehab	‘Physio and OT home service provision – I had limited funds …’ (Participant 1, 56 years old, female, 1–3 months’ stay)
Medical aid service provision and delayed transfer	‘Medical aid payment for hospital and rehab center had limited communication.’ (Participant 2, 59 years old, male, less than 1 month’s stay)
Rehab hospital	‘There was good care received at Rehab center …’ (Participant 2, 59 years old, male, less than 1 month’s stay)
Financial constraints	‘Can’t pay my medical aid in full so I can’t go to physio, I don’t have the money to do it so I can’t go there to rework my leg and that stuff or get expertise on it.’ (Participant 3, 43 years old, male, 3–6 months’ stay) ‘I had to stop that because I’m unemployed you know, and it was expensive.’ (Participant 1, 56 years old, female, 1–3 months’ stay)

**TABLE 4:** Participant responses relating to their perceived level of disability. Overarching theme: Level of disability and its influence on physical function recovery.

Sub-themes	Quotes
Emotional health	‘My chronic depression flared up significantly and impacted recovery …’ (Participant 7, 19 years old, male, 1–3 months’ stay)
No impact on recovery	‘Nothing in the hospital really impacted necessarily in a bad way to my recovery.’ (Participant 7, 19 years old, male, 1–3 months’ stay)
Negative body image	‘Now I have got a big, gaping bald spot on my head. I went to a plastic surgeon for Road Accident Fund as well and that is so damaged that hair implants won’t even work.’ (Participant 1, 56 years old, female, 1–3 months’ stay)
Perception of complications	‘If they could have done it a little bit quicker my hand will still be fine. Now my hand is not working properly.’ (Participant 8, 45 years old, male, 1–3 months’ stay)
Functional and activity limitation and functional limitations	‘I can’t yet use my arm, I can only move it a little because it can only move a little, also only just at short angles.’ (Participant 7, 19 years old, male, 1–3 months’ stay)

### Level of disability

The overarching theme from participants’ perceptions of their recovery was the impact of disability on regaining physical function. Subthemes that emerged included negative body image, physical limitations and emotional health. While some participants felt they had achieved a normal and full recovery (Participants 1 and 4), others felt they had overcome shortcomings in the medical care they received (Participants 2, 5, and 6).

Participants’ emotional state played a significant role in recovery, with personal drive helping some to overcome physical challenges (Participant 4). Challenges reported included coping with repeated surgeries (Participant 3), poor nursing care triggering previous depression (Participant 3) and the need for professional psychological support to aid recovery (Participant 7). The trauma of the accident itself was also reported to affect rehabilitation (Participants 6 and 7). In cases of incomplete recovery (Participant 5), participants experienced job loss, inability to perform certain physical activities (Participant 3), or the need to adapt how they carried out tasks (Participant 2). Personal body image was negatively affected in one participant (Participant 8).

### Pain and physical function recovery

Participants reported that both acute and chronic pain impacted physical functioning as seen in Table 3. In the acute phase after injury, a participant reported that pain had hindered physical functioning in the hospital bed (Participant 8). In severe cases of high pain levels, physical functioning was limited because of fear of pain (Participant 2). In the chronic phase after injury, the effects of pain hindered physical function from intimacy with one’s partner to activities of daily living (Participants 6, 7, 8 and 9). One participant reported no pain (Participant 5), which aided the recovery process, while another used the presence of pain as a motivator to do their ward exercise programme to improve recovery (Participant 4).

Medication was used to support the recovery of physical function; however, withdrawal symptoms following cessation were reported to hinder progress (Participants 1, 2 and 7). Persistent pain was also linked to disrupted sleep patterns, which in turn negatively affected functional outcomes. This is summarised in Table 5.

**TABLE 5:** Participant responses relating to pain and physical function recovery.

Sub-themes	Quotes (participant identification, age [years], sex, length of stay range [months])
**Overarching theme: Impact of pain**	
Pain severity	‘And you know there was a lot of times you were still in pain so you couldn’t do a lot, you had to do every single thing really slowly and you had to do it or just watch out for yourself if you don’t get hurt or stuff like that.’ (Participant 1, 56 years old, female, 1–3 months)
Fear	‘That made me so scared to bend my knee because my knee was the major problem. That made me so scared to even let a physio touch me for I think it was two days after that incident I refused that a physio come near me or touch me.’ (Participant 2, 59 years old, male, less than 1 month)
Withdrawal symptoms	‘I had withdrawal symptoms after stopping pain meds which delayed recovery.’ (Participant 7, 19 years old, male, 1–3 months)
Motivation	‘I felt pain made me stronger…’ (Participant 4, 21 years old, male, 1–3 months)

### The role of support structures in recovery of physical function

Participants consistently expressed gratitude to family, friends and neighbours (Participants 1–8) for their care and support both in-hospital and after discharge, highlighting their role in aiding the recovery of physical function. Rehabilitation centres were also described as very supportive (Participant 9) as seen in Table 6. Conversely, depression was reported to reduce motivation to work on physical recovery (Participant 3). A lack of support, particularly in-hospital, led to significant frustration (Participants 3, 6, 7 and 8). In some instances, this frustration stemmed from a perceived lack of medical support during hospitalisation (Participant 8), with participants believing that better support could have resulted in improved physical outcomes (Participants 7 and 8).

**TABLE 6:** Participant responses relating to the role of their support structures during recovery of physical function.

Sub-themes	Quotes (participant identification, age [years], sex, length of stay range [months])
**Overarching theme: The role of support structures in recovery of physical function**	
Gratitude	‘My family, friends and neighbours were strong support network.’ (Participant 7, 19 years old, male, 1–3 months) ‘My family support and funds in the medical aid for continued rehabilitation assisted a lot with recovery.’ (Participant 8, 45 year old, male, 1–3 months)
Empowerment	‘Well I would say it helped me in a way mentally you know the way they treated me … they actually mentally they spoke to me and mentally they made me strong. Without it I was never going to walk again. I thought oh, I would never be able to use my arm again you know.’ (Participant 4, 21 years old, male, 1–3 months)

An example of this is a patient who mentioned the delay in the referral process between the physiotherapist and occupational therapist to assist with upper limb function and rehabilitation to facilitate hand function (Participant 6).

### Mental health status and mental health resilience

Table 4 shows how participants described posttraumatic stress disorder symptoms, feelings of terror, hallucinations, disorientation, nightmares (Participants 4, 7 and 8), Additionally, the inability to allow others physical contact with the injury sight (Participant 7), residual pain and feelings of body disfigurement (Participant 3) were reported. The need to seek medical intervention for such symptoms (Participant 7) as seen in Table 7. Although pain was a limiting factor and recovery took noticeably long (Participants 2 and 7), limited work reintegration (Participants 2 and 6) or even inability to return to work (Participants 5, 7 and 8) was preceded by fear of ability to return to work (Participant 7). Participants reported difficulty reintegrating into the community because of both physical injuries and poor mental health (Participants 3 and 8).

**TABLE 7:** Participant responses relating to their experience of the impact of mental health of physical function recovery.

Sub-themes	Quotes (participant identification, age [years], sex, length of stay range [months])
**Overarching theme: Mental health status and mental health resilience**	
Post traumatic stress disorder	‘I struggled with nightmares long after discharge …’ (Participant 4, 21 years old, male, 1–3 months) ‘I well I was basically terrified for about 3 or 4 days after being discharged from there [*ICU*]. These hallucinations for like 2 weeks. Didn’t even know where I was.’ (Participant 8, 45 years old, male, 1–3 months)
Limited reintegration	‘I can’t work anymore as I cannot drive all day as [I was able to do] pre-injury.’ (Participant 5, 28 years old, male, 1–3 months)
Lack of return to work	‘I’m not sure if I will be able to do the work at [*deleted for the sake of anonymity*] or if I will lose that job, or what is going to happen.’ (Participant 7, 19 years old, male, 1–3 months)

ICU, intensive care unit.

## Discussion

The demographics reflected in the participants’ profiles align with those reported in other studies, with a predominance of male adults under the age of 59 (Zaidi et al. 2019; Dhaffala et al. 2013). In our study, as well as in research conducted by Seedat et al. (2009) and David (2022), male participants who experienced polytrauma had longer hospital stays than female participants, likely as a result of more severe injuries, including injuries to the lower extremities. While all participants in our study sustained injuries through motor vehicle accidents, Milton, Engelbrecht and Geyser (2021) reported varied mechanisms of injury, including falls and pedestrian accidents. Our study focused specifically on adults with multiple orthopaedic injuries who had been discharged from the hospital for 6 months to 1 year to allow for sufficient healing prior to assessment. Most participants reported difficulty reintegrating into work and the need to adapt their functionality in the workplace following the injury. This aligns with Khan, Amatya and Hoffman (2012), who similarly found a high demand for work adaptation post-injury. Moreover, our study observed that older patients experienced longer in-hospital stays, consistent with findings by Hedinger et al. (2016) and Zaidi et al. (2019).

Our study also revealed a notable link between participants’ perceptions of care and their perceived recovery post-injury. Higher satisfaction with the care received was associated with a greater sense of physical recovery, in agreement with Calydon, Robinson and Alridge (2017) and Kimmel et al. (2016), who reported a strong association between patient satisfaction and recovery outcomes. Furthermore, a patient-centred approach appeared to enhance participants’ perceptions of recovery, as participants reported feeling empowered when included in decision-making regarding rehabilitation interventions. This is supported by Kimmel et al. (2016) and Boulding et al. (2011), who observed improved compliance and encouragement when patients were actively involved in care decisions. Environmental factors, including the hospital’s appearance and noise levels, also influenced perceived recovery. Gustavson et al. (2021) similarly demonstrated that a pleasant and comfortable hospital environment positively affected functional recovery outcomes, while Tronstad et al. (2021) highlighted that non-personalised environments often contributed to negative patient perceptions.

Focusing on a private trauma rehabilitation setting in Johannesburg is relevant for several reasons. Private hospitals in this context often serve patients who have access to extended rehabilitation resources and a structured continuum of care, which allows for a detailed exploration of patient perceptions of recovery and care. Although perceptions from private healthcare patients may differ from those in public settings, they remain generalisable to the study’s aims, which focus on understanding patient-reported experiences and functional reintegration post-trauma. Insights from this setting provide valuable information on rehabilitation outcomes, patient empowerment, and environmental influences on recovery, which are critical for guiding interventions in both private and potentially public contexts.

Another finding was the discrepancies reported between day and night shift healthcare staff service delivery. Four participants expressed noise and sleep disturbances because of staff causing loud noises and providing poor nursing care, which was also seen by Palese et al. (2017), who conducted a secondary analysis of 12 Italian nursing units and also found that patients were not satisfied with nursing care as a result of noise disturbances. The majority of participants reported that physiotherapy played a major role in their functional recovery, which has been shown by Silvester, Trompeter and Hing (2021) as well. Despite this positive perception of physiotherapy, some participants felt that the high turnover of physiotherapy staff often limited progress because of a lack of continuity of care. Participants also reported that they perceived better recovery outcomes when they received positive reinforcement and efficient communication. This is in concordance with findings by Jensen (2026), who found that patient’s reported better recovery outcomes when there was good communication between the patient and healthcare practitioners.

The socioeconomic relationship with access to rehabilitative care cannot go unnoticed. Participants reported reduced affordability of rehabilitation services upon discharge, which resulted in their perceptions of poorer functional outcomes. This issue is further compounded by the fact that medical aid funders often do not adequately cover rehabilitative care, frequently resulting in substantial out-of-pocket expenses to the patients themselves. Allen et al. (2022) highlighted that affordability remains a significant barrier to recovery. Because of the high levels of out-of-pocket expenditure on rehabilitation, compounded with the high levels of job losses after injury, return to work becomes that much harder for patients. This aligns with findings by Wyse et al. (2020) who reported that there is a reduced rate of return to work in patients who sustained injuries from motor vehicle accidents because of poor recovery outcomes associated with the lack of affordability of rehabilitation services.

A factor often overlooked in physical recovery is the role that mental health plays in perceptions of recovery, where participants express that depression and lack of motivation hinder progress. Participants reported that there was inadequate mental health support provided to them in the trauma unit during their hospital stay, which they felt impacted their functional outcomes. Similarly, Wiseman, Foster and Curtis (2013) reported a lack of mental health referral and follow up for their patients who sustained polytrauma injury and its negative impact on their recovery outcomes. Participants in the current study reported experiencing physical limitations such as pain, restricted mobility, and poor quality of life after polytrauma, which Schneiderman, Van Aswegen and Becker (2013) also observed in their study of trauma survivors when assessed for quality of life at 6 months after hospital discharge.

A relevant finding of our study is that pain in the acute phase of recovery limited participants’ functional recovery because of the fear of pain and movement. Nummela et al. (2022) and Shafeeq et al. (2022) reported a high correlation between pain and recovery, with pain often being a barrier to movement. An unexpected finding was the impact of pain during intimacy, which negatively affected some participants’ quality of life and underscored the need for a holistic approach to rehabilitation. Effective pain management and interprofessional collaboration are essential for recovery following polytrauma. Another finding of the current study highlighted delays in referral between physiotherapy and occupational therapy for upper limb rehabilitation, which prolonged pain and hindered functional recovery. These challenges align with literature emphasising the importance of interprofessional rehabilitation in optimising service delivery and patient outcomes (Keller 2024). Strengthening collaboration frameworks can improve access to timely interventions, enhancing both pain management and hand function rehabilitation.

Family support was reported to be a major facilitator of functional recovery, consistent with findings by Critchfield et al. (2019) and De Beer and Brysiewicz (2017). These authors highlighted the importance of strong support networks in improving functional outcomes for patients who sustained polytrauma injuries. Additionally, the patient-practitioner relationship affected recovery by improved outcomes associated with positive relationships, echoing the research of Khan et al. (2012), who conducted a prospective cross-sectional study in patients with polytrauma and traumatic brain injury, and found that the relationship with the healthcare provider was a predictor of recovery outcomes.

### Strengths and limitations

The strength of our study lies in its qualitative approach, addressing the gap in understanding what patients experience and perceive in their recovery of function following polytrauma injury. These qualitative insights highlighted barriers and facilitators to recovery, offering valuable clinical implications. Our study also has limitations, such as a small sample size from one facility in Gauteng and the high potential for recall bias. Additionally, the reflexivity and positionality of the principal author, a rehabilitation professional, may have influenced how data was perceived. Also, potential bias from purposive sampling and self-reported data, limited exploration of mental health factors, and a narrow temporal scope may restrict the generalisability and depth of the findings.

## Conclusion

Our study revealed six key themes which affect the recovery of physical function of adults following polytrauma injury in the Gauteng region of South Africa. Positive mental health and strong relationships with healthcare providers were associated with improved perceived outcomes, while physical limitations, pain, financial constraints and poor quality of care received hindered perceptions of recovery. Our study findings may inform rehabilitation clinical practice as well as clinical guidelines to advocate for improved affordability of rehabilitation services to improved functional recovery outcomes. This research should be repeated on a broader and more inclusive scale, beginning with an assessment of whether public health patients have similar perceptions. Further research is required to map referral pathways to mental health practitioners from patient admission to hospital discharge to post-discharge follow up. In addition, patients’ recovery outcomes need to be monitored to identify issues in recovery as they arise.
